# Supplementary material for: Hierarchical classification of immune cell transcriptomes at population-scale
Source: bioRxiv. 2026 Jun 2:2026.05.30.728980. Preprint. [Version 1] doi: 10.64898/2026.05.30.728980 (PMC13252358; doi:10.64898/2026.05.30.728980)
Supplement: Supplementary file 2 [file media-2.pdf]

Supplementary Figures | Beltz et. al.

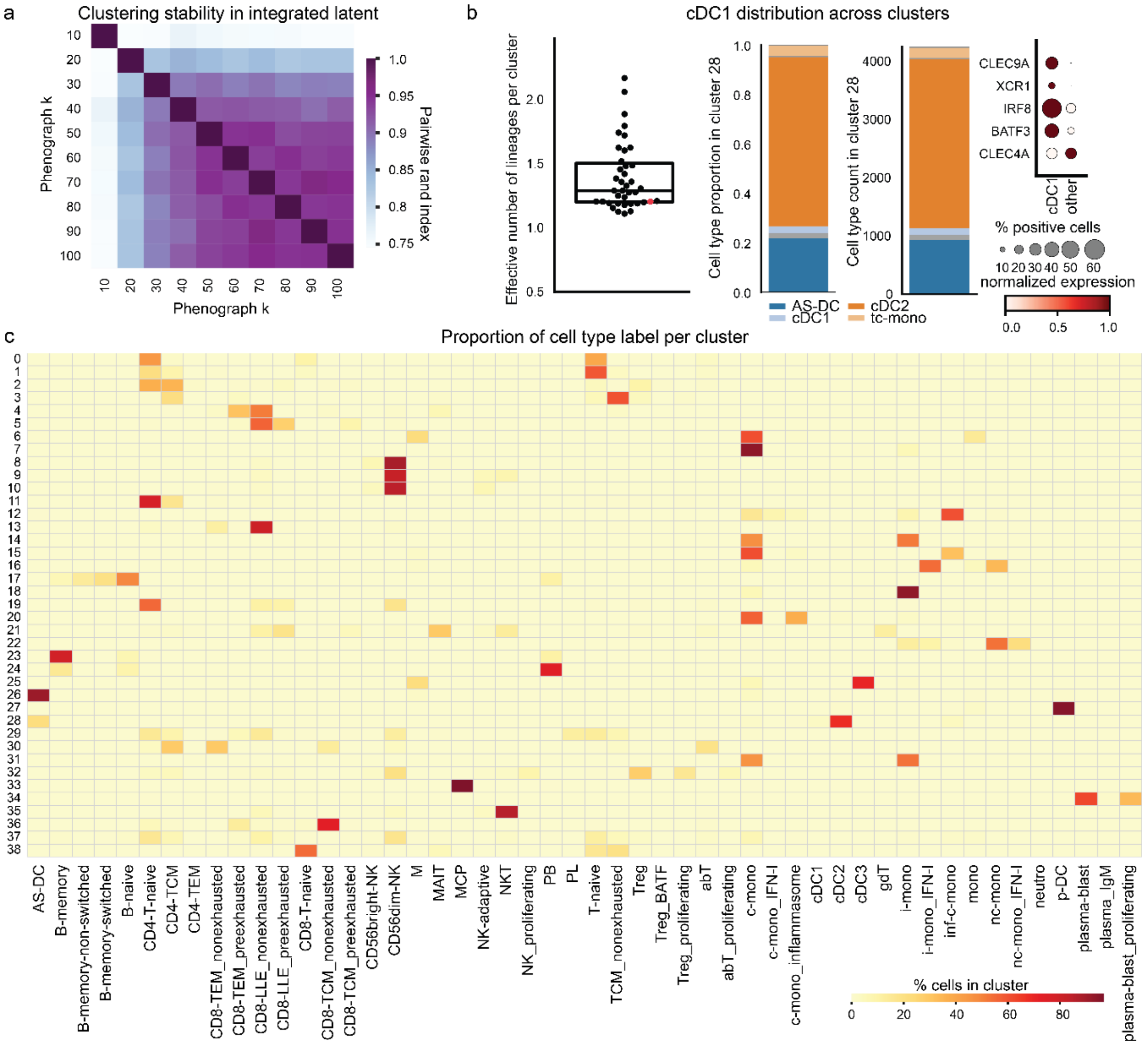

Supplementary Data Fig. 1 | **Rare cell type clusters are lost during unsupervised data integration| a**, We integrated a 4-dataset subset of Suco PBMC resource (Methods) using scVI with 30 latent dimensions on 2000 highly variable genes. We clustered cells using Phenograph and identified k=30 as the minimum k providing stable clusters as shown by the pairwise rand index. **b**, While conventional dendritic cells type 1 (cDC1) fall into a cluster with a low degree of mixing as determined by the effective lineage number (left plot in red), they only represent a minority of the cell types in this cluster (bar graphs, middle) Other cells in the cluster did not show cDC1 marker expression confirming that cDC1 would be lost in unsupervised data integration. **c**, This heatmap of cell type proportion per cluster indicates a large number of mixed clusters. tc: transitional classical, mono: monocyte, NK: Natural Killer, MAIT: mucosa-associated invariant chain T cell, AS DC (AXL & SIGLEC6 positive dendritic cell), baso (basophil), cDC (conventional dendritic cell), HSC (hematopoietic stem cell), MAIT (mucosa associated invariant chain T cell), mono (monocyte), NK (Natural Killer), tc (transitional classical), B (B cell), c mono (classical monocyte), DN (double negative), eosino (eosinophil), ery (erythrocyte), exh. (exhausted), gdT (gamma delta T cell), gran (granulocyte), IFN (interferon), ILC (innate lymphoid cell), i mono (intermediate monocyte), M (myeloid cell), nc mono (non classical monocyte), neutro (neutrophil), PB (plasma or B lineage), prol. (proliferating), T (T cell), TCM (T central memory), tc-mono (transitional classifcal monocyte) TEM (T effector memory), term. (terminally), TNK (T or innate lymphoid cell), Treg (regulatory T cell).

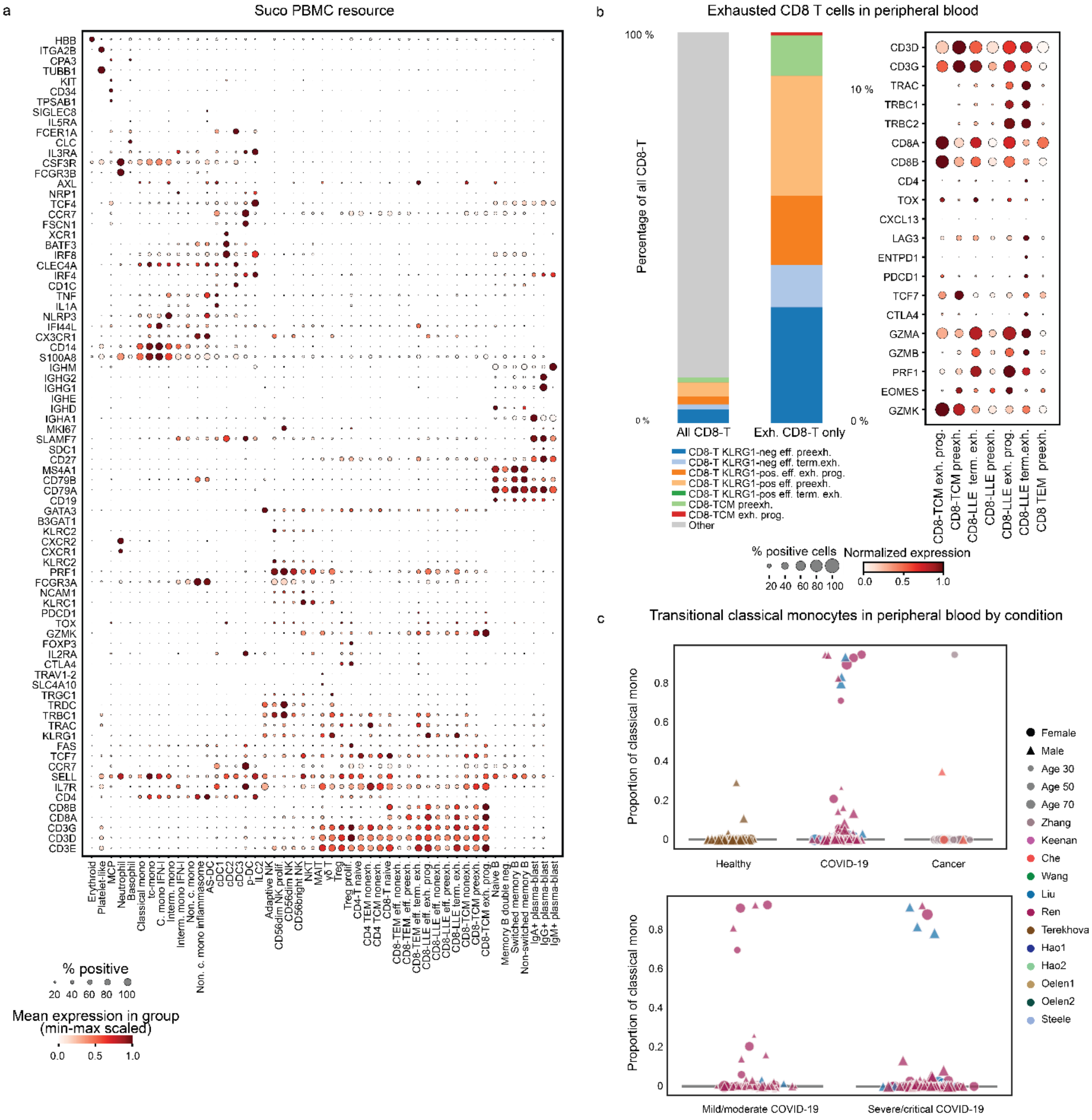

Supplementary Data Fig. 2 | Cell types in the Suco peripheral blood mononuclear cells resource | a, Dotplot showing marker gene expression leaf-node labels from our Suco [eripheral blood mononuclear cell (PBMC) resource (n= 5,389,645 cells). Traitional classical monocytes (tc-mono) show clear monocyte identity and express no dendritic cell markers. B (B cell), c mono (classical monocyte), DN (double negative), eosino (eosinophil), ery (erythrocyte), exh. (exhausted), gdT (gamma delta T cell), gran (granulocyte), IFN (interferon), ILC (innate lymphoid cell), i mono (intermediate monocyte), M (myeloid cell), nc mono (non classical monocyte), neutro (neutrophil), PB (plasma or B lineage), prol. (proliferating), T (T cell), TCM (T central memory), tc-mono (transitional classifcal monocyte) TEM (T effector memory), term. (terminally), TNK (T or innate lymphoid cell), Treg (regulatory T cell). **b**, Stacked barplots showing the proportions of exhausted relative to all CD8-T cells in our Suco PBMC atlas, dotplot showing the expression patterns of these exhausted CD8-T cell subsets. Only, CD8 LLE span the entire spectrum of exhaustion states exh.: exhausted, term.: terminally, preexh.: preexhausted, prog.: progenitor . c, Transitional classical monocytes are conserved across a multitude of health states, ages, datasets, both in male and female donors, as shown in a dotplot highlighting the proportion relative to all classical monocytes per sample.

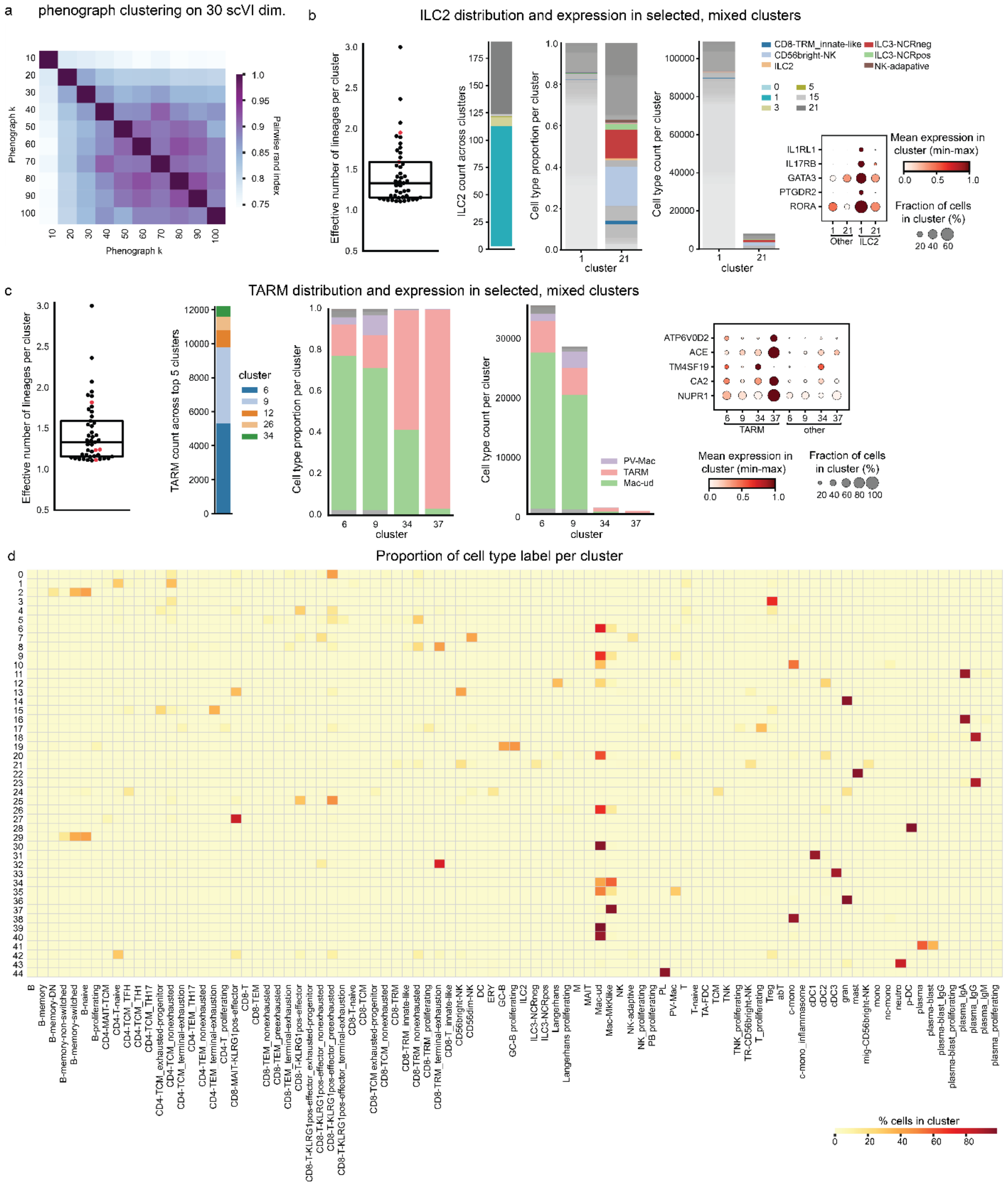

Supplementary Data Fig. 3 | Granular cell types are lost in unsupervised data integration | a, We integrated our Suco TIL data using scVI with 30 latent dimensions on 2000 highly variable genes (Methods). We clustered cells using Phenograph and identified k=60 as the minimum k providing stable clusters as shown by the pairwise rand index. **b**, Label homogeneity as judged by the effective number of lineages per cluster shows how innate lymphoid cells type 2 (ILC2) are assigned to mixed clusters 1 and 21 (left plot, in red). ILC2s are distributed across several clusters (2nd plot from the left), and even in the clusters containing the most ILC2, they co-occur with other cell types (3rd and 4th plot from left). Cell type marker gene expression (plot on the right) confirmed that the remaining cells in the clusters were not of ILC2 lineage. **c**, Many tumor-associated resorptive macrophages (TARM) clusters (in red) showed strong cell type mixing (plot on the left). Clusters 6 and 9 contained most TARM (2nd plot from the left), but are dominated by other macrophage subtypes (3rd plot from the left). Other cells in the cluster generally lacked TARM marker gene expression confirming that TARM will be confused with other cell type during unsupervised data integration. **d**, This heatmap of cell type proportion per cluster indicates a large number of mixed clusters.

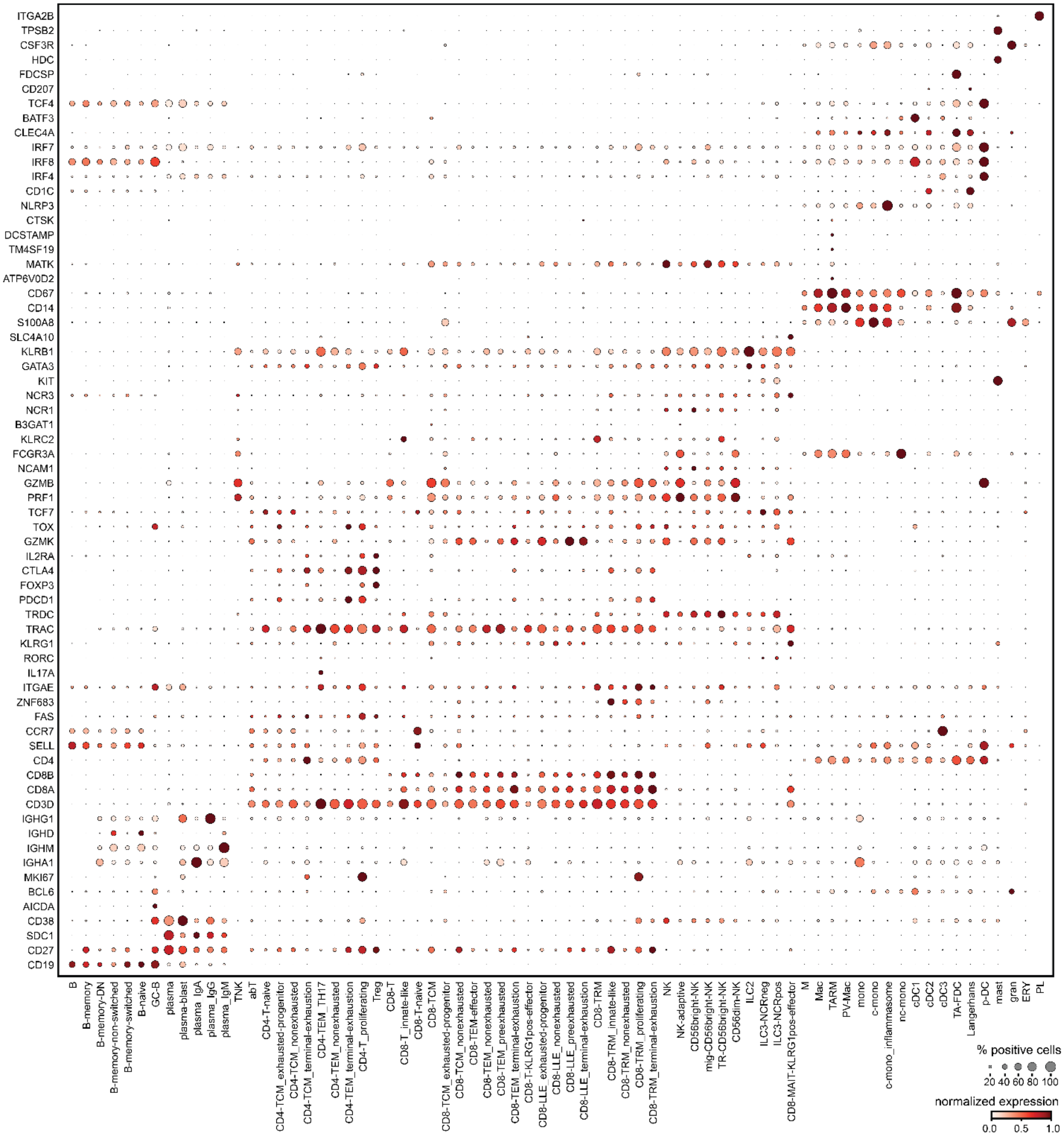

Supplementary Data Fig. 4 | Expression patterns in tumor infiltrating leukocytes | Dotplot showing expression patterns of different cell types across in our Suco tumor-infiltrating leukocyte (TIL) resource (n=764,571 cells). CD56bright NK cells frequently show a tissue resident phenotype marked by ITGAE and ZNF683 alongside an absence of SELL. TARM lacked canonical dendritic cell markers (CD1C, CD1A, BATF3, IRF7, IRF4, IRF8). c- (classical), cDC (conventional dendritic cell), DN (double negative), exh (exhausted), GC (germinal center B cells), gran (granulocyte), ILC (innate lymphoid cell), innate like (innate like T cells), LLE (long lived effector), M (myeloid), Mac (Macrophage), MAIT (mucosa associated invariant chain T cells), mem (memory), mono (monocyte), nc (non classical), neutro (neutrophil), NK (Natural Killer cell), ns (non switched), PB (plasma or B cell lineage), plasma (plasma cell), prog (progenitor), prol. (proliferating), PV (perivascular), sw. (switched), T (T cell), TA FDC (tumor associated follicular dendritic cell), TARM (tissue associated resorptive macrophages), TEM (T effector memory), TH17 (T helper 17), TNK (T or ILC lineage).

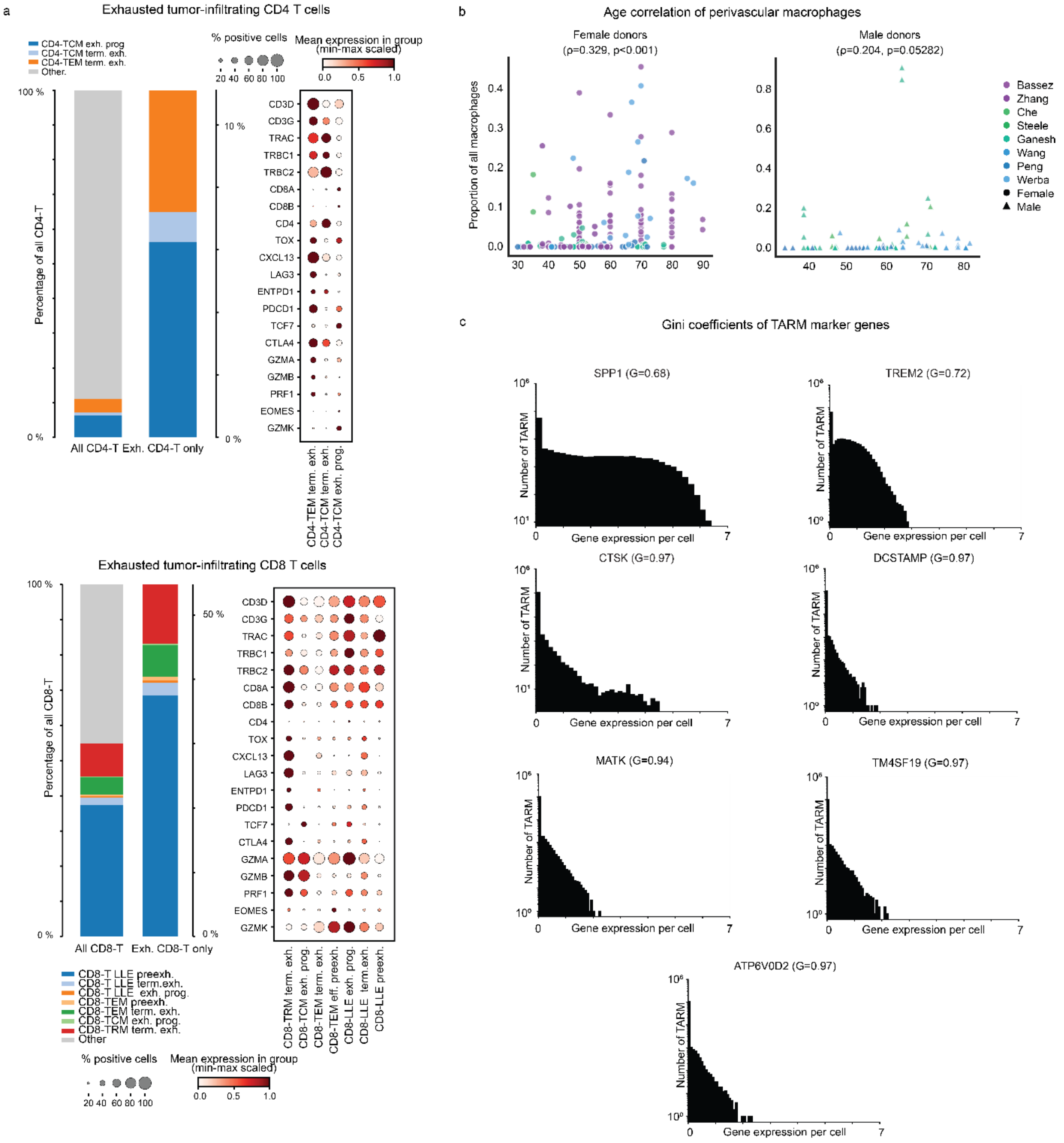

Supplementary Data Fig. 5 | Tumor infiltrating leukocytes in Suco **a**, Stacked barplots showing the proportions of exhausted relative to all CD4-T/CD8-T cells in our Suco TIL atlas, dotplot showing the normalized marker gene expression of these exhausted subsets. TRM: tissue-resident memory T cells, TCM: central memory T cells, LLE: long-lived effector T cells, term.: terminally, exh.: exhausted, prog.: progenitor, preexh.: pre-exhausted. **b** Dotplots showing the correlation between the proportion of perivascular relative to all tumor-infiltrating macrophages by age, dataset and sex with statistically significant positive correlation between age and the PV-Mac proportion in female donors. Correlations were quantified using the Pearson correlation coefficient. **c**, Distributions of normalized, log1p-transformed gene expressions in TARM with the Gini coefficient provided per gene. TARM-exclusive genes such as ATP6V0D2, CTSK, DCSTAMP, MATK, and TM4SF19 show high Gini coefficients, contrasting with the more homogeneous expression of classical macrophage markers such as SPP1 and TREM2.

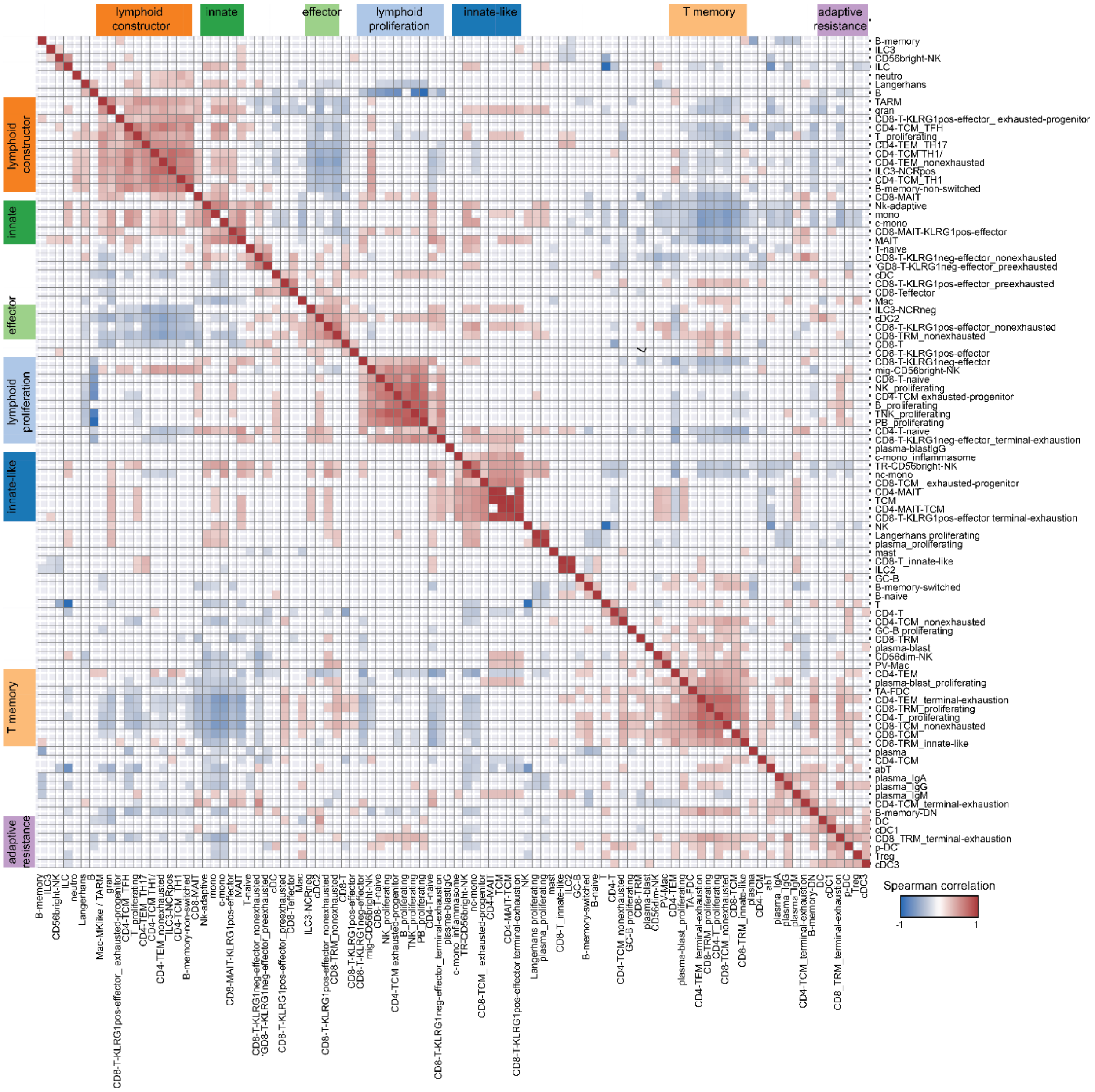

Supplementary Data Fig. 6 | Cell type co-occurence identifies tumor immune microenvironments | We quantified cell type co-occurence by pairwise spearman rank correlation based on cell type frequency in our Suco TIL atlas (n=310 patient samples, Methods). This uncovered thetumor immune microenvironments (TIME) indicated with a color bar.

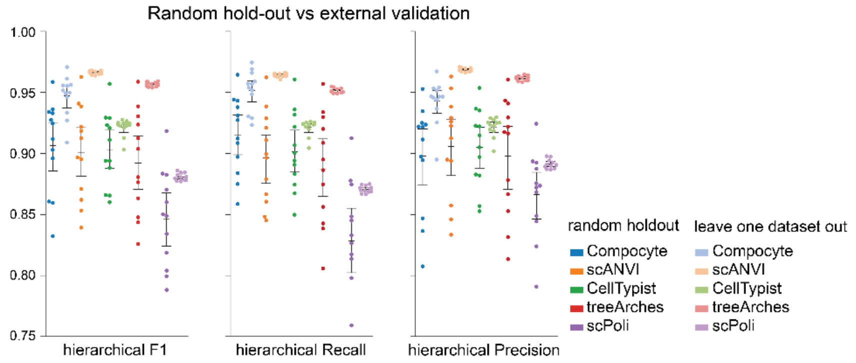

Supplementary Data Fig. 7 | Cell-level holdout overestimates generalization ability | For the five Python methods we benchmarked, we simulated equivalent experiments by holding out dataset-sized randomly selected holdout data. In all methods, mixed-dataset holdouts (lighter colors) overestimated the ability of classifiers to generalize, shown by systematically higher performance benchmarks compared to dataset-level holdouts (darker colors).

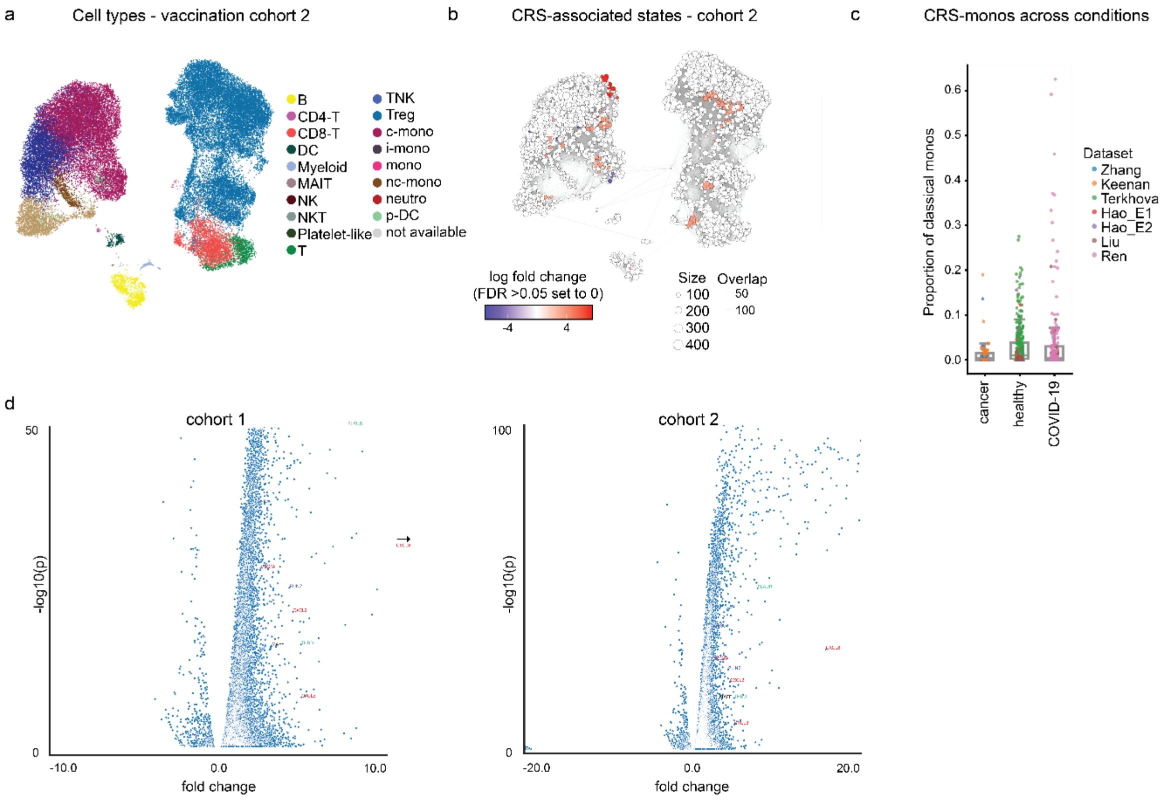

Supplementary Data Fig. 8 | Validation of subclinical cytokine release syndrome-associated monocytes in an independent cohort | a, UMAP showing cell types in cohort 2 of patients (n=6) receiving a SARS-CoV-2 vaccine during ICT. This data was used for testing of the subclinical cytokine release syndrome associated monocyte (CRS-mono) classifier trained on cohort 1. b, Specific monocyte neighborhoods (CRS-monos) were significantly associated with high CRS-associated cytokine levels as revealed by Milo (false discovery rate (FDR)=0.05). c, Using our expanded classical monocyte sub-classifier, we transferred CRS-mono labels to 7 PBMC datasets totaling 775 samples across a total of 389 healthy donors, patients with cancer and active infectious disease, finding high proportions of CRS-monos particularly in patients suffering from COVID-19. d, Volcano plots show consistenly upregulated CRS-mono associated genes in cohort 1 and cohort 2 as compared to other classical monocytes with p values calculated using Mann-Whitney-U tests. Genes with a higher fold change outside of the axis are indicated with an arrow.

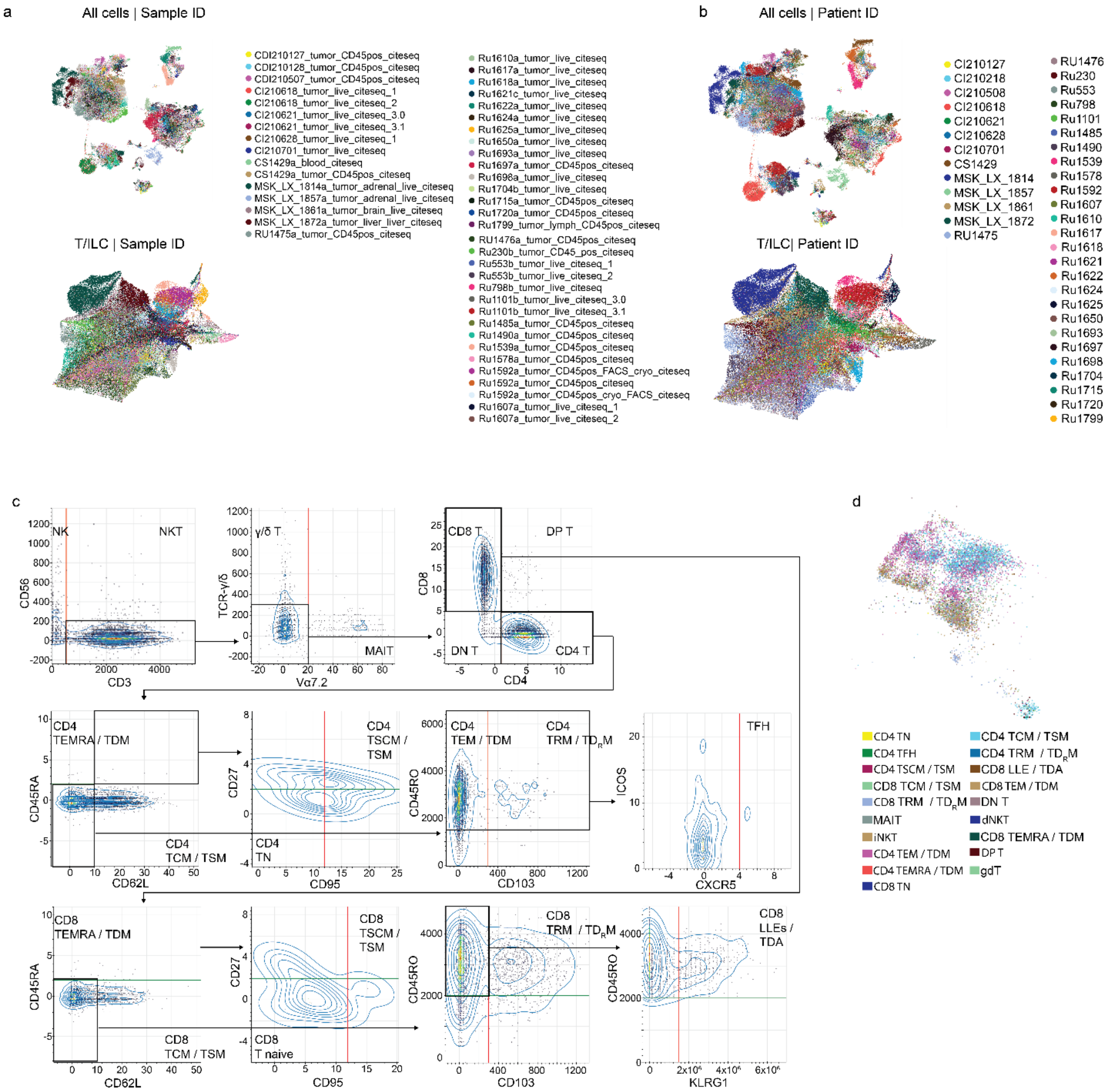

Supplementary Data Fig. 9 | CITEseq analysis of tumor infiltrating leukocytes | a, UMAP embeddings of tumor infiltrating leukocytes (n=150,059) or tumor infiltrating leukocytes (n=84,222) colored by sample or by **b**, patient. **c**, T and innate lymphoid cells in CITE-seq samples were manually gated per sample according to the indicated gating strategy on DSB antibody-derived tag data. NK: Natural Killer cell, NKT: Natural Killer T cell, TEMRA: Effector memory RA T cell, TCM: Central memory T cell, TSCM: Stem cell memory T cell, LLE: Long-lived effector cell, TRM: Tissue-resident memory T cell. We also indicate a modular T cell nomenclature: A: activated, D: disseminated, S: Secondary lymphoid organ homing, M: memory, R: resident. Mean Spectra cell score per cell type and sample in metastases versus primary tumors in our pancreatic and lung cancer cohort (n=39 patients). **b**, Frequencies of indicated memory subsets in tumor types from the Human Tumor Atlas Network (HTAN) cohort (n=160 patients). **c**, Mean gene program scores in biopsies from metastatic and primary tumors across cancer types (breast cancer (BRCA), colorectal adenocarcinoma (COAD), lung adenocarcinoma (LUAD), small cell lung cancer (SCLC)) in the HTAN cohort (n=160 patients). All p values were calculated using Mann-Whitney U tests with significant comparisons highlighted with an asterisk according to the Benjamini-Hochberg method.

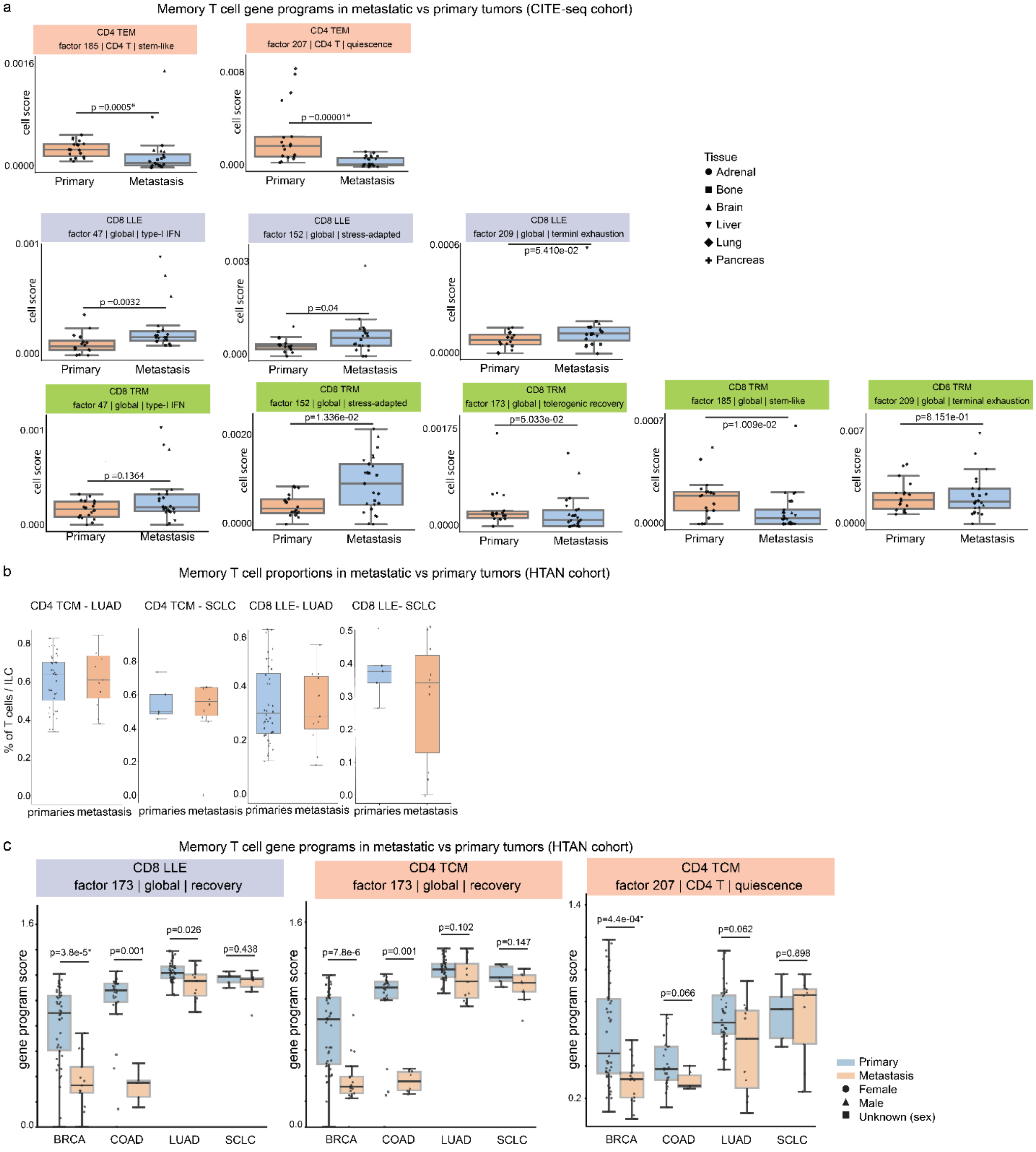

Supplementary Data Fig. 10 | Gene expression programs in memory T cells across tumor types | a, Mean Spectra cell score per cell type and sample in metastases versus primary tumors in our pancreatic and lung cancer cohort (n=39 patients). **b**, Frequencies of indicated memory subsets in tumor types from the Human Tumor Atlas Network (HTAN) cohort (n=160 patients). **c**, Mean gene program scores in biopsies from metastatic and primary tumors across cancer types (breast cancer (BRCA), colorectal adenocarcinoma (COAD), lung adenocarcinoma (LUAD), small cell lung cancer (SCLC)) in the HTAN cohort (n=160 patients). All p values were calculated using Mann-Whitney U tests with significant comparisons highlighted with an asterisk according to the Benjamini-Hochberg method.

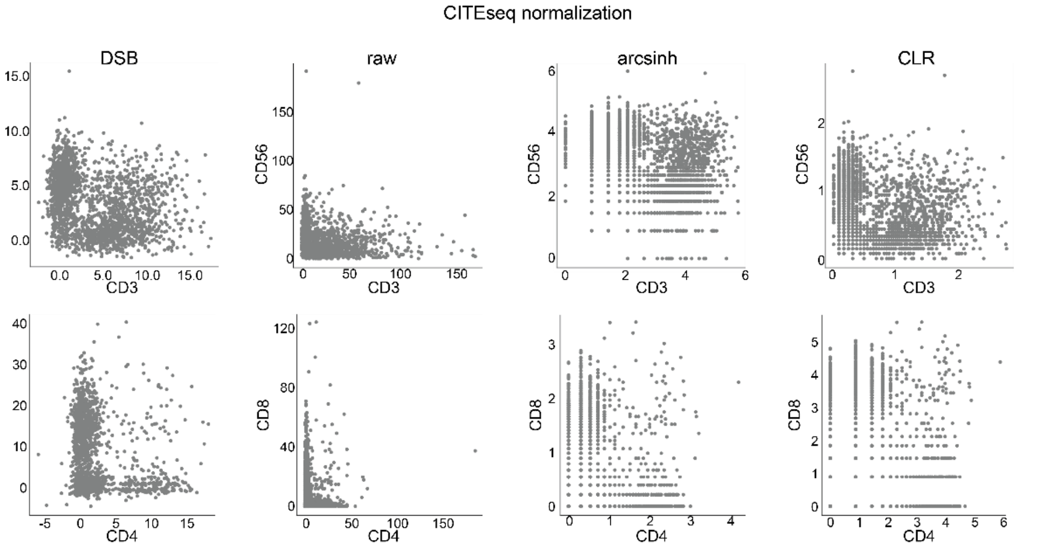

Supplementary Data Fig. 11 | Normalization of CITE-seq data | Comparison of immune cell subset separation by different CITEseq normalization methods (raw, DSB normalization, arcsinh normalization, CLR normalization) shows DSBs ability to identify major immune cell lineages (n=1 representative patient)
